# Membrane vesicle-mediated release of bacterial RNA

**DOI:** 10.1038/srep15329

**Published:** 2015-10-20

**Authors:** Annika E. Sjöström, Linda Sandblad, Bernt Eric Uhlin, Sun Nyunt Wai

**Affiliations:** 1 Department of Molecular Biology, Umeå University, S-90187 Umeå, Sweden; 2 The Laboratory for Molecular Infection Medicine Sweden (MIMS), Umeå University, S-91807, Sweden

## Abstract

Many Gram-negative bacterial species release outer membrane vesicles (OMVs) that interact with the host by delivering virulence factors. Here, we report for the first time that RNA is among the wide variety of bacterial components that are associated with OMVs. To characterize the RNA profiles of bacterial OMVs, we performed RNA deep sequencing analysis using OMV samples isolated from a wild type *Vibrio cholerae* O1 El Tor strain. The results showed that RNAs originating from intergenic regions were the most abundant. Our findings reveal a hitherto unrecognised feature of OMVs mimicking eukaryotic exosomes and highlight a need to evaluate the potential role of RNA-containing bacterial membrane vesicles in bacteria-host interactions.

Outer membrane vesicles (OMVs) are constantly being discharged from the surface of Gram-negative bacteria during growth. OMVs typically range between 50–200 nm in diameter and contain outer membrane proteins, LPS, phospholipids, and some periplasmic constituents^1^.

Studies of bacterial OMVs have revealed some similarities with vesicles known as exosomes that are secreted by most mammalian cell types. OMVs and exosomes have similar size ranges and both carry payloads of proteins, lipids, and genetic material enclosed in membrane-bound spherical structures. Both types can deliver functional molecules to distant extracellular compartments and tissues. Exosomes are involved in: horizontal transfer of genetic material^2^; transport of mRNAs and miRNAs from mast cells to recipient mast cells^3^; delivery of miRNAs from T cells to antigen presenting cells to modulate gene expression^4^ and delivery of viral miRNAs from infected cells to non-infected cells^5^. These capabilities collectively suggest that exosomes, by being natural carriers and intercellular transporters of RNA, are suitable candidates for delivery of exogenous gene interfering compounds, such as siRNA.

The function of bacterial OMVs to aid in transport of a variety of bacterial products- such as proteins, enzymes, and DNA is well described. Extensive studies, reviewed elsewhere^1^,^6^,^7^, have proven the importance of a vesicle-mediated transport system and clearly describe the ways in which different bacteria make use of this delivery system. Several virulence-associated factors of pathogenic bacteria can be detected in association with OMVs^8^,^9^,^10^. OMVs likely facilitate direct delivery of virulence factors to target host cells rather than to the external milieu where diffusion could lessen the functional impact of the molecules.

Their similarity to eukaryotic exosomes and the fact that OMVs act as transporters of various bacterial constituents prompted us to investigate if an “exosome-like” RNA delivery system by bacterial OMVs exists.

## Results and Discussion

### Detection of RNA associated with *V. cholerae* A1552 vesicles

**Figure 1:**
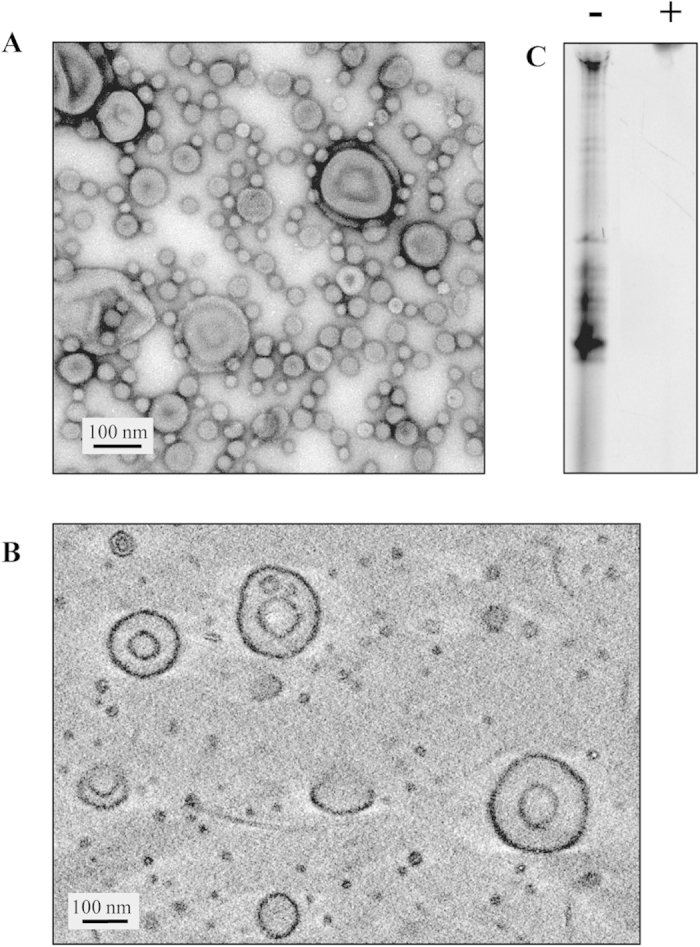
*Vibrio cholerae* O1 El Tor A1552 membrane vesicles. (**A**) Negative staining micrograph of purified vesicles before hot phenol treatment. Bar: 100 nm. (**B**) Cryo-electron tomogram of purified vesicles. The image shows a projection of 10 sections corresponding to 6 nm in the centre of the tomogram volume. (**C**) Polyacrylamide denaturing gel (12%) with 10 μg DNase-treated samples after (–); No RNase treatment, (+); RNase treatment.

OMVs from the wild type *V. cholerae* strain A1552 were isolated as described earlier^11^,^12^. The ultrastructural analysis of OMVs was performed using transmission electron microscopy (TEM) and cryo-electron tomography. As shown in Fig. 1A, we observed OMVs with diameters ranging from 25–200 nm by TEM. The 3D volume of the purified vesicles was analysed by cryo-electron tomography and interestingly revealed that larger vesicles comprised double membranes (Fig. 1B). Morphologically these OMVs may appear similar to bacterial minicells which are achromosomal particles derived from bacterial cells with mutations inactivating genes controlling normal bacterial cell division^13^. However, OMVs are released during normal growth of bacteria and the *V. cholerae* strain used has no aberration of the normal process of cell division. Furthermore, the OMVs are distinctly smaller in size in comparison with minicells.

In order to investigate if OMVs may contain RNA, vesicle samples from *V. cholerae* strain A1552 were subjected to hot phenol RNA extraction and purification (Materials and Methods). After two DNase treatments, the sample was dissolved in DEPC-H_2_O. Two 10 μg-samples were prepared where one sample was subjected to RNase treatment. Both samples were run on a 12% denaturing PAGE gel, GelRed stained and visualized by UV light (Fig. 1C). The result strongly suggested that the separated bands from the un-treated sample mainly consisted of RNA since no bands in the RNase-treated sample were detected.

The OMV samples were prepared from dense overnight cultures of *V. cholerae* strain A1552 since the amount of OMVs obtained depends on the amount of bacteria used and gave a higher yield in comparison with OMV isolation from the exponentially growing bacteria. However, we also tested whether RNA can be observed in association with OMVs isolated from the exponential growth phase of bacteria. As shown in Supplementary Fig. S1A, lane 2, RNA was also associated with OMVs isolated from the exponential growth phase of bacteria. It suggested that the release of RNA from the bacterial cells in association with OMVs was not growth phase dependent. Furthermore, OMV-associated RNA was not likely due to contamination by nucleic acids from some lysis of bacterial cells. A live/dead staining analysis was performed for the estimation of the fraction dead bacterial cells as described in the Materials and Methods.

There was no apparent difference in the ratio of live and dead cells in bacterial populations when the log-phase bacterial culture and overnight culture were compared and in both cases there were very few dead cells detected (Supplementary Fig. S1C). Moreover, by Western immunoblot analysis we examined the possible presence of a cytoplasmic protein (RpoS) in samples of the culture supernatant before and after ultracentrifugation, and in OMV samples. As shown in Supplementary Fig. S1B, there was no RpoS detected in the supernatant and OMV samples although RpoS was observed in the whole cell lysate sample. The results indicated that there was little or no lysis of bacteria causing contamination of cytoplasmic components in the culture supernatants.

To clarify whether or not the detected RNA was tightly associated with the vesicles and not simply appeared there due to co-precipitation of somehow released RNA, e.g. from lysed bacterial cells during ultracentrifugation steps, OMVs were purified using an Optiprep density gradient centrifugation procedure as described in the Materials and Methods. Five distinct rings (fractions) could visually be detected in the centrifuged tube (Supplementary Fig. S2A, left panel). All five fractions were collected for further analyses. The presence of OMVs in these fractions was monitored by immunoblotting using polyclonal antiserum raised against the OmpU protein which is a major component of OMVs from *V. cholerae* strains and can be used as an OMV marker^12^. The OmpU protein was detected in the fractions 3, 4, and 5 indicating that these fractions contained OMVs (Supplementary Fig. S2A, right panel). RNA extraction was performed with each of the fractions and the presence of RNA was analysed after electrophoresis on a 12% denaturing PAGE gel by GelRed staining and visualization under UV light. As shown in Supplementary Fig. S2B, left panel, an appreciable amount of RNA was detected in the fraction 3 and a lesser amount was observed in the fraction 4 whereas there was no detectable RNA in the other fractions (Supplementary Fig. S2B, left panel, lanes 1 + 2, 3 and 4).

We concluded from these tests that RNA indeed was associated tightly with OMVs.

### Sequence analysis of vesicle RNA

As a mean to directly identify the RNA detected in vesicles, we performed sequencing using MiSeq (Illumina) of rRNA-depleted RNA preparations obtained from the vesicles released by strain A1552. The RNA isolation kit was used to acquire ultrapure RNA from which most of the rRNA was removed by the procedure of the Ribo-Zero Magnetic Kit (Epicentre) before RNA library preparation as described in the Materials and Methods. After paired-end sequencing, the resulting 80 bp sequences were aligned to the sequenced chromosomal DNA of *V. cholerae* strain O1 El Tor N16961, a strain very closely related to the *V. cholerae* strain O1 El Tor A1552. A typical alignment view using Integrative Genomics Viewer (IGV) software is shown in Supplementary Fig. S3A. The following criteria were used to determine if alignments were of interest: i) that the number of short RNA sequences aligned to a specific DNA nucleotide (hit) was more than 200; ii) that these 200 DNA nucleotide hits included a homology stretch longer than 70 nucleotides. Regions of this size are presented in Supplementary Table 1A (chromosome I) and Supplementary Table 1B (chromosome II). Table 1 summarizes the total of 308 regions that fulfil the above-described criteria. Of these regions, 76 ppm (224/2,961,149 regions/bp) belong to chromosome I, whereas 77 ppm (83/1,072,315 regions/bp) belong to chromosome II. These figures indicate that the number of regions aligned to a chromosome is proportional to the size of that chromosome. This is also the case when comparing the numbers of intergenic regions. When comparing the number of regions in RNA coding for proteins with unknown functions almost half of the regions in chromosome II codes for parts of uncharacterized proteins, in contrast to one-sixth in chromosome I (Table 1). This can be explained by the fact that only half of the proteins encoded on chromosome II are characterized, while on chromosome I around five-sixths have known functions. Interestingly, the majority of the most abundant RNA detected corresponds to non-coding regions.

Extensive studies of vesicles from Gram-negative bacteria over the last decades suggest that formation of vesicles occurs as the bacterial outer membrane creates a bleb with outer membrane proteins and lipids that surrounds the contents of the periplasm. However, a recent report by Pérez-Cruz *et al.* describes a sub-population of vesicles—outer-inner membrane vesicles (O-IMV) in which not only the outer but also the inner membrane is involved in the formation of the surface^14^. Such double-membrane vesicles would therefore have the ability to carry cytoplasmic constituents, leading to a few speculations on how RNA might enter the vesicles. One explanation might be via protein synthesis that occurs in the vicinity of the blebbing membrane. Translation machinery proteins are entrapped in vesicles of *V. cholerae*^15^ and *E. coli* vesicles^16^, thus these ribosomal proteins might simply bring their translating mRNA. Another explanation for how RNA enters vesicles is that free-floating RNA in the cytoplasm might be entrapped as well as some cytoplasmic constituents during vesicle formation. The actual localization of RNA in association with OMVs, whether or not the RNA is inside of the vesicles is as yet unclear. Attempts to use RNase treatments of OMVs as a means to reveal the localization have remained inconclusive. In our preliminary studies we observed an apparent loss of RNA after RNase treatment of OMVs. However, since it could not be determined how such treatments per se affected the integrity of the vesicles, or to what extent the RNase could enter vesicles, we need to employ additional approaches to assess the localization.

### Analysis of highly transcribed RNA regions in vesicles

Our sequencing results further prompted us to investigate if the RNA sequences most frequently found in vesicles — high-hit regions — were transcribed at levels sufficient to be detected by Northern blotting. Three different probes were constructed corresponding to regions containing the highest peaks, the Highly Transcribed RNA Regions (HTRRs). These HTRRs are: the upstream region of *vc2479* (uncharacterized protein) from chromosome I (1,090,000 hits), the intergenic region between *vc0190* (DNA helicase II) and *vc0191* (uncharacterized protein) from chromosome I (120,000 hits), and the intergenic region between *vca0526* (H^+^/Cl^-^ exchange transporter ClcA) and *vca0527* (uncharacterized protein) from chromosome II (115,300 hits) (Supplementary Fig. S3B–D). Ultrapure RNA was extracted from A1552 whole cells and from vesicles, respectively, and analysed by Northern blot. Hybridization was done with probe *vc2478,5* followed by stripping and re-probing of the same membrane using probes *vc0190,5* and *vca0526,5* (in that order). The Northern blot analysis clearly revealed that these high-hit regions were transcribed as the transcripts were detected in the whole cell RNA preparations and they were present at detectable levels in the vesicles (Supplementary Fig. S4A–D).

Furthermore, Northern blot analysis using probe *vc2478,5* on extractions from the density gradient purified vesicle fractions also showed that fractions with RNA contained HTRRs (Supplementary Fig. S2B, right panel: lanes 3 and 4). To ensure that the DNAse I treatment could remove any contaminating DNA in the RNA preparations we performed a PCR test on extracted vesicle RNA before and after DNase I treatment using primers that would recognize DNA corresponding to the HTRR upstream of *vc2479* and *vc0633*. Evidently the DNase I treatment successfully removed such DNA as no PCR bands were obtained after this treatment (Supplementary Fig. S5)

**Figure 2:**
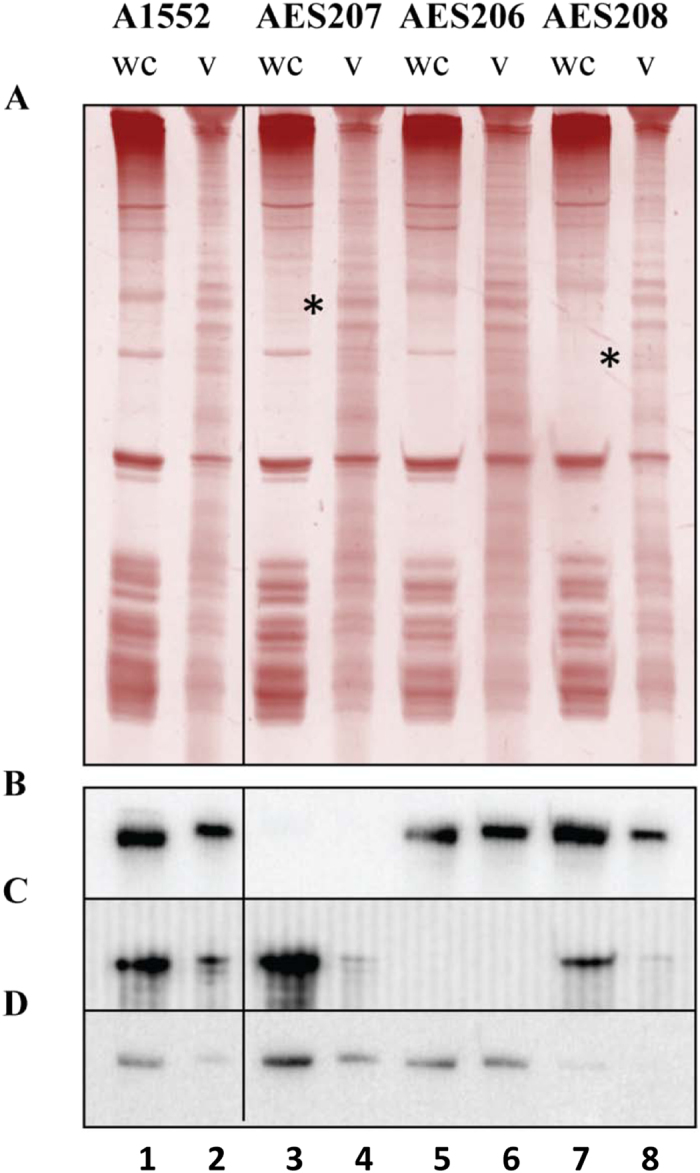
Analysis of OMV-associated RNA. RNA extracted from overnight cultures of wt and HTRR mutant *Vibrio cholerae* O1 El Tor A1552 whole cells (wc) and vesicles (v) using the Total RNA Norgen kit. RNA (3.5 μg) was run on a 12% polyacrylamide denaturing gel and transferred to a membrane. (**A**) Gel stained with GelRed. Positions of bands missing in case of mutant strains are indicated by asterisks (*). (**B**) Northern blot membrane probed with probe *vc2478,5*. (**C**) Northern blot membrane re-probed with probe *vc0190,5*. (**D**) Northern blot membrane re-probed with probe *vca0526,5*.

To verify that the vesicle RNA sequences aligned to the chromosomal *V. cholerae* DNA— corresponding to the HTRR regions — in fact give rise to the vesicle transcripts, we constructed three mutants (AES206, AES207, and AES208) in which the chromosomal DNA corresponding to each of these HTRRs was deleted and replaced by Km-cassettes. RNA from vesicle preparations and whole cell extracts of wild type A1552 and the three mutants were subjected to a 12% polyacrylamide denaturing gel electrophoresis and stained with GelRed (Fig. 2A). All vesicle-isolated RNA (v) samples exhibited a similar but unique band pattern, different from the likewise similar but unique whole cell extracted RNA (wc) sample pattern. Asterisks (*) in the AES207 and AES208 mutant lanes show where bands are missing in the samples from mutants. The gel-separated RNA transcripts were transferred to a membrane for Northern blotting and hybridized with probes *vc2478,5* (Supplementary Fig. S4B), *vc0190,5* (Supplementary Fig. S4C), and *vca0526,5* (Supplementary Fig. S4D). Probing verified that these RNAs from HTRRs correspond to the identified DNA sequences on the chromosome (Fig. 2B–D). Interestingly, the size of the bands missing in the AES207 and AES208 RNA wc lanes are the same as that of the transcripts recognized by the *vc2478,5* and *vca0526,5* probes (Fig. 2A,B and D). Thus, the *vc2478,5* and *vca0526,5* region transcripts are apparently abundant enough to be visible by GelRed staining.

As vesicles are known to be taken up by other bacteria and by eukaryotic cells^12^,^17^, we may hypothesize that such OMV-associated RNA might cause regulatory effects in the new “host” cells. OMV internalization into eukaryotic cells has been demonstrated to mediate intracellular antigen exposure and recent studies with vesicles from the bacterium *Aggregatibacter actinomycetemcomitans* showed that they acted as strong inducers of cytoplasmic peptidoglycan sensor NOD1- and NOD2-dependent NF-κB activation in human embryonic kidney cells^18^.

Interestingly, earlier studies demonstrated the successful siRNA inhibition of the *Staphylococcus aureus* coagulase gene and suggested that siRNAs could effectively modulate virulence and drug resistance of *S. aureus*^19^. Subsequent studies revealed that siRNAs-mediated regulation of recipient bacterial genes expression was dependent upon the RNA-binding protein Hfq^20^,^21^. In these studies, synthetic siRNA was introduced into the recipient bacterial cells using electroporation. We suggest that OMV-associated sRNA with the ability to modulate the expression of target genes could be introduced into the recipient bacterial cells using OMVs as a RNA delivery vehicle. From earlier studies, there is evidence that the OMV membrane can fuse with the outer membrane of a recipient bacterium and thereby introduce the OMV luminal contents into the recipient bacteria, including peptidoglycan hydrolase causing the lysis of recipient bacterial cells^22^. On the basis of earlier and present findings, we propose that it is highly likely that OMV-associated RNA can be delivered into either or both prokaryotic and eukaryotic host cells and then can cause modulation of the expression of some of their genes.

In conclusion, we report for the first time the detection and successful isolation of bacterial RNA released via OMVs from *V. cholerae*, providing a proof of principle that bacterial RNA can be released from bacteria via membrane vesicles. The origin of three selected sRNA was confirmed by Northern blotting analysis using the wild type strain and its respective mutant construct. Our findings show that we need to consider the potential role(s) of RNA-containing bacterial membrane vesicles in bacteria-host and bacteria-bacteria interactions.

## Material and Methods

### Bacterial strains, plasmids, and growth conditions

The strains and plasmids used in the present work are described in Table 2 and primers are described in Table 3. Unless otherwise stated, the strains were grown at 37 °C in either Luria–Bertani (LB) broth with vigorous shaking or on LB agar. When necessary, media was supplemented at the following concentrations: sucrose (Suc), 10% (w/v); Carbenicillin (Cb), 50 μgml^−1^; Kanamycin (Km), 25 μgml^−1^; and Rifampicin (Rif), 100 μgml^−1^.

### Construction of plasmids for Km cassette replacement mutagenesis

Mutant plasmid constructs were designed with a 1500 bp Km-cassette flanked by 500-bp sequences corresponding to upstream and downstream regions of the target gene. The Km-cassette and flanking regions co-amplified as one unit by PCR. The upstream (A and B primers) and downstream (C and D primers) flanking regions were PCR amplified (Kapa HiFi HotStart Polymerase, Kapa Biosystems) from A1552 chromosomal DNA and cloned into pJET1.2 for sequencing and amplification purposes. Correct pJET1.2 constructs were digested with *Xho*I and *Xba*I and the desired fragments were gel purified. The Km-cassette was PCR amplified from plasmid pKD4 (FRTup and FRTdo primers, DreamTaq Green PCR Master Mix (Fermenta)) and gel-purified. Fifty to 90 ng of the flanking region DNA and 200 ng of the Km-cassette DNA were used for cross-over PCR (Kapa HiFi HotStart Polymerase, Kapa Bisystems) with the following conditions: anneal 43 °C–20 s, elongation 69 °C–1.45 min). The reaction was purified on a gel where the resulting 2500-bp fragments were excised and cloned into pJET1.2 for sequencing and amplification purposes. Correct constructs were digested with *Nru*I and the gel-purified fragments were cloned into *Sma*I-digested pCVD442 yielding plasmids pCVD-190,5::Km, pCVD-2478,5::Km, and pCVD-526,5::Km.

### Genetic manipulation to obtain Km replacement mutants

Wild type *V. cholerae* A1552 (recipient) was cross streaked with SM10λ/pCVD-190,5::Km, SM10λ/pCVD-2478,5::Km, and SM10λ/pCVD-526,5::Km (donors), respectively, on non-selective LB plates and incubated at 37 °C for 6 h. A1552/pCVD-construct integrants (cross sections) were then selected for on LB + Km + Rif plates. To cure the integrants of transfer plasmids and to select for homologous recombination of Km-cassette replacement mutations, Km^R^ + Rif^R^-colonies were suspended in LB, serially diluted, and spread on LB + Km + Suc plates. Successful mutagenesis of plasmid-free (Cb^S^) Km-cassette replacement clones was verified by PCR using AA and DD primers. Mutagenesis gave rise to strains AES206 (A1552Δ*vc0190* - *vc0191*), AES207 (A1552Δ*vc02478 - vc2479*), and AES208 (A1552Δ*vca0527* - *vca527*).

### Vesicle isolation

OMVs were isolated from bacterial culture supernatants essentially as described previously^8^. Briefly, bacteria were inoculated in 1 litre flasks containing 280 ml LB. Cultures were grown with shaking at 37 °C for 16 h and then centrifuged at 8,250 × *g* for 15 min at 4 °C. Pellets were discarded and supernatant re-centrifuged twice. This supernatant was filtered twice through vacuum driven 0.2 μm pore size stericup filters (Millipore Express™ Plus) and ultra-centrifuged at 100,000 × *g* for 2 h at 4 °C in a 45 Ti rotor (Beckman). The vesicle pellets were re-suspended in 600 μl TE buffer and kept at −80 °C until further use.

### Density gradient centrifugation

Isolated vesicles suspended in TE buffer were fractionated by the density gradient centrifugation method slightly modified from previously described^12^. Shortly, a gradient of Optiprep (Sigma-Aldrich) was created in a 4 ml ultracentrifugation tube (0.4 ml 45%, 0.5 ml 35%, 0.6 ml 30%, 0.6 ml 25%, 0.6 ml 20%, 0.5 ml 15% and 0.6 ml 10%). The vesicle sample was added and the tube was centrifuged (180, 000 × *g*, 3 h, 4 °C) in a SW60Ti rotor (Beckman). After centrifugation visual fraction bands were extracted (100–300 μl), washed in 60 ml TE buffer, and ultra-centrifuged at 100,000 × *g* for 2 h at 4 °C in a 45 Ti rotor (Beckman). The resulting fraction pellets were re-suspended in 20–80 μl TE buffer. 1 μl of the suspended fractions were taken for immunoblot analysis with anti OmpU and the rest kept at −80 °C until further use.

For tests with RNase treatment, vesicles obtained from an overnight culture were suspended in 300 μl TE buffer and 50 μg RNase A (QIAgen) was added. The sample was kept at RT for 15 min, washed in 70 ml TE buffer, and ultra-centrifuged at 100,000 × *g* for 2 h at 4 °C in a 45 Ti rotor (Beckman). The resulting vesicle pellet was re-suspended in 60 μl SUPERase• In™ RNase Inhibitor buffer (20 mM Tris-HCl, pH 7.5, 50 mM NaCl, 1 mM EDTA) and 100U inhibitor (Life Technologies) was added before analysis.

### Live/dead staining and fluorescence microscopy analyses

Live/dead staining was performed using the LIVE/DEAD® BacLightTM Bacterial Viability Kit L13152 according to the manual. In short, *V. cholerae* O1 El Tor strain A1552 cultures grown to OD_600_ = 0.7 (log-phase) or OD_600_ = 4.6 (overnight culture) were harvested by centrifugation and suspended in 0.85% NaCl. After additional centrifugation and washing, bacterial suspensions of 10^6^ bacteria/ml were subjected to staining as described by the manufacturer (Molecular Probes). Treatment of a sample with isopropanol was done to obtain a population of only dead bacteria for comparison. The staining was examined and visualized in a Nikon fluorescence microscope with bandpass filters for Texas Red and Fluorescein, respectively.

### Electron microscopy

For negative staining 3.5 μl of sample was adsorbed for 2 min onto glow-discharged formvar and carbon-coated copper grids, washed in H_2_O and immediately negatively stained in 50 μl of 1.5% uranyl acetate solution for 30 s. Negative-stained samples were examined on a JEOL JEM1230 TEM operating at 80 kV. Micrographs were recorded with a Gatan MSC 600CW CCD camera using Digital Micrograph software. For cryo-electron tomography 4 μl sample was vitrified on a holey carbon film using FEI Vitrobot. The tomography data was collected on a JEOL 2200FS TEM with SerialEM software and a pixel size of 0.57 nm at the Caesar institute in Bonn, Germany. The 3D reconstruction was performed with SIRT and visualized using the IMOD package from University of Colorado at Boulder^23^.

### RNA extraction

RNA was extracted from whole cells (1 ml culture) or vesicles isolated from 2.5–5 litre cultures. Extraction was initially made by modified hot phenol extraction methods described by von Gabain *et al.*^24^. Briefly, vesicles were disrupted in 3% SDS followed by RNA hot phenol extraction. After DNase I (Fermenta) treatment, phenol::chloroform purification, and ethanol precipitation the resulting RNA pellet was dissolved in 100 μl DEPC-treated water.

Less yield but cleaner RNA was obtained using the Total RNA Purification Plus Kit (Norgen Biotek corp) according to the manufacturer’s manual followed by treatment with DNase I. RNA was kept at −80 °C until further use.

To check if the DNase I treatment efficiently removed any contaminating DNA, the RNA preparations were subjected to PCR amplification using DreamTaq Green PCR Master Mix (2×) (Fermenta) and two primer pairs, ompU1up + ompU3do and vc2478,5up + vc2478,5do, respectively. The templates for each set were 100ng vesicle RNA isolated with the Norgen kit and samples without or with DNase I treatment were tested.

### RNA preparation for the library construction

Isolated vesicle RNA was treated with the Ribo-Zero Magnetic Kit for Gram-Negative Bacteria (Epicentre) according to the manufacturer’s instructions to reduce the amount of rRNA. This rRNA-depleted RNA was used for library construction using the TruSeq RNA sample prep kit v2 (Illumina) according to the manufacturer’s protocol (#15026495 revD).

Briefly, the rRNA-depleted RNA was fragmented and the RNA was reverse transcribed into cDNA using random-sequence primers and SuperScript III reverse transcriptase. The cDNA was purified using AMPure XP beads (Beckman Coulter) and amplified for 15 cycles of PCR with barcoded primers, followed by purification using AMPure beads.

### Cluster generation and sequencing

An 18-pM solution of RNA was subjected to cluster generation and paired-end sequencing with 80-bp read length on the MiSeq system (Illumina Inc.) using the v3 chemistry according to the manufacturer’s protocols.

Base calling was done on the instrument by RTA 1.18.42 and the resulting .bcl files were demultiplexed and converted to fastq format with tools provided by CASAVA 1.8.2 (Illumina Inc.), allowing for one mismatch in the index sequence. Additional statistics on sequence quality were compiled with an in-house script from the fastq-files, RTA and CASAVA output files. Sequencing was performed by the SNP&SEQ Technology Platform in Uppsala, Sweden www.sequencing.se.

### RNA sequencing analysis

RNA sequences were aligned to the N16961 *Vibrio cholerae* chromosomes using Integrative Genomics Viewer (IGV) software^25^,^26^

### Polyacrylamide gel electrophoresis (PAGE) and Northern blot analyses

Whole cell RNA and vesicle RNA from the strains were separated on a 12% acrylamide:bis-acrylamide 19:1/8 M urea gel. The gel was stained with GelRed dye (Biotium) for visualization and then the RNA was transferred onto an Amersham Hybond™-XL membrane (GE Healthcare) for 1 h at 120 mA, 15 V on a semi-dry transfer unit (EBU-4000, C. B. S Scientific). The blotted membrane was cross-linked with UV. 30 ng of specific probes generated by PCR were [α-^32^P]ATP-labelled using the Prime-a-Gene^®^ Labeling System (Promega) according to the manufacturer’s instructions. Unincorporated nucleotides were removed with MicroSpin™ G-25 Columns (GE Healthcare). Prehybridization (4 h) and hybridization (16 h) was performed in Roti-Hybri Solution (Roth) at 58 °C. Bands were visualized using a Phospho-Imager, Storm 860 (Molecular Dynamics).

Stripping of the membrane was done by 2 × 15 min washing on shaker at RT in Wash 1 (0.01 M EDTA, 0.05 × SSC, 0.1% SDS—heated to 100 °C) followed by 2 × 15 min washing on shaker at RT in Wash 2 (0.02 × SSC).

### SDS-PAGE and Western blotting analyses

Protein extracts were separated by SDS-polyacrylamide gel electrophoresis and thereafter transferred to a polyvinylidene fluoride (PVDF) micro-porus membrane. OmpU polyclonal antibodies were used to detect outer membrane constituents (vesicles) in the Optiprep fractions. RpoS was used as possible cytoplasmic hallmark for lysed bacteria in the supernatants recognizable by anti-RpoS polyclonal antibodies. Anti-rabbit HRP-conjugated antibody (Agrisera) was used as secondary antiserum at a final dilution of 1:25,000. Further visualization was carried out using Clarity^TM^ Western ECL substrate as described by the manufacturer (Bio-Rad). Bands were detected using the ChemiDoc XRS system (Bio-Rad).

## Additional Information

**How to cite this article**: Sjöström, A. E. *et al.* Membrane vesicle-mediated release of bacterial RNA. *Sci. Rep.* **5**, 15329; doi: 10.1038/srep15329 (2015).

## Supplementary Material

Supplementary Information

## Figures and Tables

**Table 1:** Summary of OMV RNA sequence alignments to the *V. cholerae* N16961 genome.

	Chromosome I	Chromosome II	Bothchromosomes
Size	2961149 bp	1072315 bp	4033464 bp
Size/Total size	73.4%	26.6%	100%
Regiona)	224	83	307
Region/Total size	76 ppm	77 ppm	76 ppm
Intergenic regionb)	11	3	14
Intergenic region/Region	4.9%	3.6%	4.5%
Up- or downstream region	18	13	31
Up- or downstream region/Region	8.0%	15.7%	10.1%
Uncharacterizedc)	38	38	74
Uncharacterized/Region	17.0%	45.8%	24.1%
Characterizedd)	157	29	186
Characterized/Region	70.1%	34.9%	60.6%
Membrane associatede)	70	13	83
Membrane associated/Region	31.1%	15.7%	27.0%
Membrane associated/Characterized	44.6%	44.8%	44.6%
Cytoplasmicf)	73	12	85
Cytoplasmic/Region	32.6%	14.4%	27.7%
Cytoplasmic/Characterized	46.5%	41.4%	45.6%
sRNA	17	11	28
sRNA/Region	7.6%	13%	9.1%

^a)^Region: RNA-aligned DNA stretch >70 nucleotides and >200 hits.

^b)^Intergenic region: region situated downstream of both flanking genes.

^c)^Uncharacterized: region within an uncharacterized protein coding sequence.

^d)^Characterized: region within a characterized protein coding sequence.

^e)^Membrane associated: region within a membrane associated protein coding sequence.

^f)^Cytoplasmic: region within a cytoplasmic protein coding sequence.

**Table 2:** Strains and plasmids used in this study.

Strain/Plasmid	Description/Relevant characteristics	Reference/Source
*V. cholerae* Strains		
A1552	O1 El Tor, Inaba, Rif^R^	27
A1552∆*rpoS*	∆*rpoS* derivative of A1552	28
AES206	A1552, replacement of inter-genic region *VC0190* - *VC0191* with Km-cassette	This study
AES207	A1552, replacement of upstream region of *VC2479* with Km-cassette	This study
AES208	A1552, replacement of inter-genic region *VCA0526* - *VCA0527* with Km-cassette	This study
*E. coli* Strains		
SM10λpir	*thi thr leu tonA lacY supE recA*::RP4-2 Tc::Mu Km λpir	29
Plasmids		
pJET1.2	Cloning vector, Cb^R^	Fermenta
pKD4	Carrying frt-flanking Km-cassette	30
pCVD442	Suicide vector, Cb^R^	31
pCVD-190,5::Km	pCVD442 carrying Δvc*0190-1*::Km	This study
pCVD-2478,5::Km	pCVD442 carrying Δvc*2478-9*::Km	This study
pCVD-526,5::Km	pCVD442 carrying Δvca*0526-7*::Km	This study

**Table 3:** Primers used in this study.

Primer name	Primer sequence (5′ to 3′)	Bold letters
*Primers for cloning*		
FRTup	GTGTAGGCTGGAGCTGCTT	
FRTdo	CATATGAATATCCTCCTTAG	
vc2478upA	GC**TCGCGA**CAGTTGGTGCTTAGCTTTTGC	*Nru*I site
vc2478doB	**CTAAGGAGGATATTCATATGA**GGGTAAATCAATGCTGTGTTTATTGC	Complement to FRTdo
vc2479upC	**GAAGCAGCTCCAGCCTACAC**GGGGTTCTCTCTATGACAGATTCTCG	Complement to FRTup
vc2479doD	GC**TCGCGA**GCAGTTGTTCGACTTGTTGGC	*Nru*I site
vc2478upAA	CCAATCCTACCACTTCATTGCC	
vc2479doDD	GTAACGGAATATCCCAGCTTGC	
vc0190upA	GC**TCGCGA**AAGCTCTACATCACCTACG	*Nru*I site
vc0190doB	**CTAAGGAGGATATTCATATG**CGAGTAAGACTCTAACCCC	Complement to FRTdo
vc0191C	**GAAGCAGCTCCAGCCTACA**CGAAATTTTGAACA	Complement to FRTup
vc0191D	GCTCGCGACTTCACAAAAGGCATGATGA	*Nru*I site
vc0190upAA	GAAGAGGCGGGGCGTCTCGAAG	
vc0191DD	CAAAAAAACGCTAGCACACCGACGC	
vca0526A	GC**TCGCGA**CCATTTGTACTGTAGGACCTTC	*Nru*I site
vca0526B	**CTAAGGAGGATATTCATATG**CCAATTCATCAATAGGTG	Complement to FRTdo
vca0527upC	**GAAGCAGCTCCAGCCTACAC**GGATATACTTCGGCCAAAAGG	Complement to FRTup
vca0527doD	GC**TCGCGA**CACTGATCGACTTTGTTCCCC	*Nru*I site
vca0526AA	GCGCGTATCTTCATTTTTGACACG	
vca0527doDD	GGCATCAATGTCACTACTGCTG	
*Primers for Northern blot probes*		
vc2478,5up	CCCTGGGGTGTTCGTCAGCGGAT	
vc2478,5do	CGTTGAGGATCGTAGCCCTTGAAC	
vc0190,5up	CCTTGATAGTACGCTGCAATCCGTC	
vc0190,5do	GGACATTGAACGGACGCAATCG	
vca0526,5up	GATGTGCCGGAATTATACGCTGC	
vca0526,5do	GGTTCCATATCCTCCATCAGAAATG	
ompUup1	GCGTCGACGCTTGATGCATCACCTATTTCG	
ompUdo3	GTCAACACGGTCTGCTACAGC	
