# Supplementary material for: Membrane vesicle-mediated release of bacterial RNA
Source: Sci Rep. 2015 Oct 20;5:15329. doi: 10.1038/srep15329 (PMC4612299; doi:10.1038/srep15329)
Supplement: Supplementary Information [file srep15329-s1.pdf]

Supplementary Information:

Membrane vesicle-mediated release of bacterial RNA

Annika E. Sjöström[1,2], Linda Sandblad[1,2], Bernt Eric Uhlin[1,2], Sun Nyunt Wai[1,2] *

[1]Department of Molecular Biology, Umeå University, S-90187 Umeå, Sweden

[2]The Laboratory for Molecular Infection Medicine Sweden (MIMS), Umeå University, S-91807, Sweden

* Corresponding author: Tel: +46-90-7856704,

E-mail: sun.nyunt.wai@molbiol.umu.se

Legends to Supplementary figures

Supplementary Data Fig. S1.

A) Analyses of RNA extracted from a log-phase (OD = 0.7) culture of *Vibrio cholerae* O1 El Tor strain A1552. Left panel: RNA extracted from whole cells (lane 1) and vesicles (lane 2) using the Total RNA Norgen kit. 1.5 μg RNA were run on a 12 % polyacrylamide denaturing gel stained in GelRed (light red). Right panel: RNA was transferred to a membrane for Northern blot analysis and probed with a probe for gene *vc2478,5* (lane 3; whole cells and lane 4: vesicles)

B) Western blot analysis of using antibodies recognizing RpoS to check for possible lysis of bacteria. Lane 1: whole cell extract from overnight culture; lane 2: supernatant from overnight culture after filtration through 0.22 μm stericup filters; lane 3: supernatant from overnight culture after ultracentrifugation; lane 4: vesicles from overnight culture; lane 5: whole cell extract from a culture at OD_{600} = 3 and lane 6: whole cell extract from A1552 RpoS mutant.

C) Fluorescence microscopy analysis of bacteria after live/dead staining. Staining was performed using the LIVE/DEAD® BacLightTM Bacterial Viability Kit L13152 with bacterial samples from *V. cholerae* O1 El Tor strain A1552 cultures as follows: a) sample from OD_{600} = 0.7 (log-phase); b) sample from OD_{600} = 4.6 (overnight culture); c) sample from OD_{600} = 4.6 (overnight culture) after treatment with isopropanol to obtain a population of only dead bacteria for comparison.

Supplementary Data Fig. S2. Density gradient centrifugation purification of vesicles from an overnight culture of the *V. cholerae* O1 El Tor strain A1552. **A.** Left panel: Photo of the

density gradient tube after ultracentrifugation of a vesicle sample. The five visible rings (fractions) are numbered 1 to 5 in increasing density order. Right panel: Western blot analysis of samples from each numbered fraction using antibodies recognizing OmpU (VC 0633, Outer membrane protein U, 37kDa).

B. Analyses of RNA extracted from each of the five density gradient fractions in A. RNA extracts obtained using the Total RNA Norgen kit were separated on a 12 % polyacrylamide denaturing gel. The gel was stained and subsequently blotted for transfer to a membrane. Left panel: Gel stained with GelRed. Right panel: Northern blot membrane probed with probe *vc2478,5*. Lanes: wc, whole cell RNA extract; 1 - 5: samples originating from each of the numbered density gradient fractions described in A.

Supplementary Data Fig. S3. Illustrations of vesicle RNA (isolated from *V. cholerae* strain A1552) sequences aligned to chromosomal DNA sequences of *V. cholerae* N16961 strain. Red alignments are in the sense direction, blue alignments are in the anti-sense direction. *: Height indicates the number of sequences aligned. **A.** Typical alignment view **B.** Region *vc2478 – vc2479* **C.** Region *vc0190 – vc0191* **D.** Region *vca0526 – vca0527*.

Supplementary Data Fig. S4. RNA extracted from an overnight culture of *Vibrio cholerae* O1 El Tor A1552 whole cells (wc) and vesicles (v) using the Total RNA Norgen kit. RNA from whole cells (5 μg) and RNA from vesicles (3.5μg) were run on a 12 % polyacrylamide denaturing gel stained in GelRed (panel A). The RNA was transferred to a membrane that was probed with probe *vc2478,5* (panel B) and consecutively re-probed first with probe *vc0190,5* (panel C) and then with probe *vca0526,5* (panel D).

Supplementary Data Fig. 5. DNase I treatment of RNA extracted from bacterial vesicles. PCR analysis for detection of DNA in RNA samples extracted from vesicles released by *Vibrio cholerae* O1 El Tor strain A1552. The analysis was performed before (lanes 2 & 3) and after (lanes 4 & 5) treatment with DNase I. PCR products after amplifications using *ompU* primers (lanes 2 & 4) or *vc2478,5* primers (lanes 3 & 5) were analysed on a 1% agarose gel. Lane 1 shows a GeneRuler™ DNA Ladder (Fermentas).

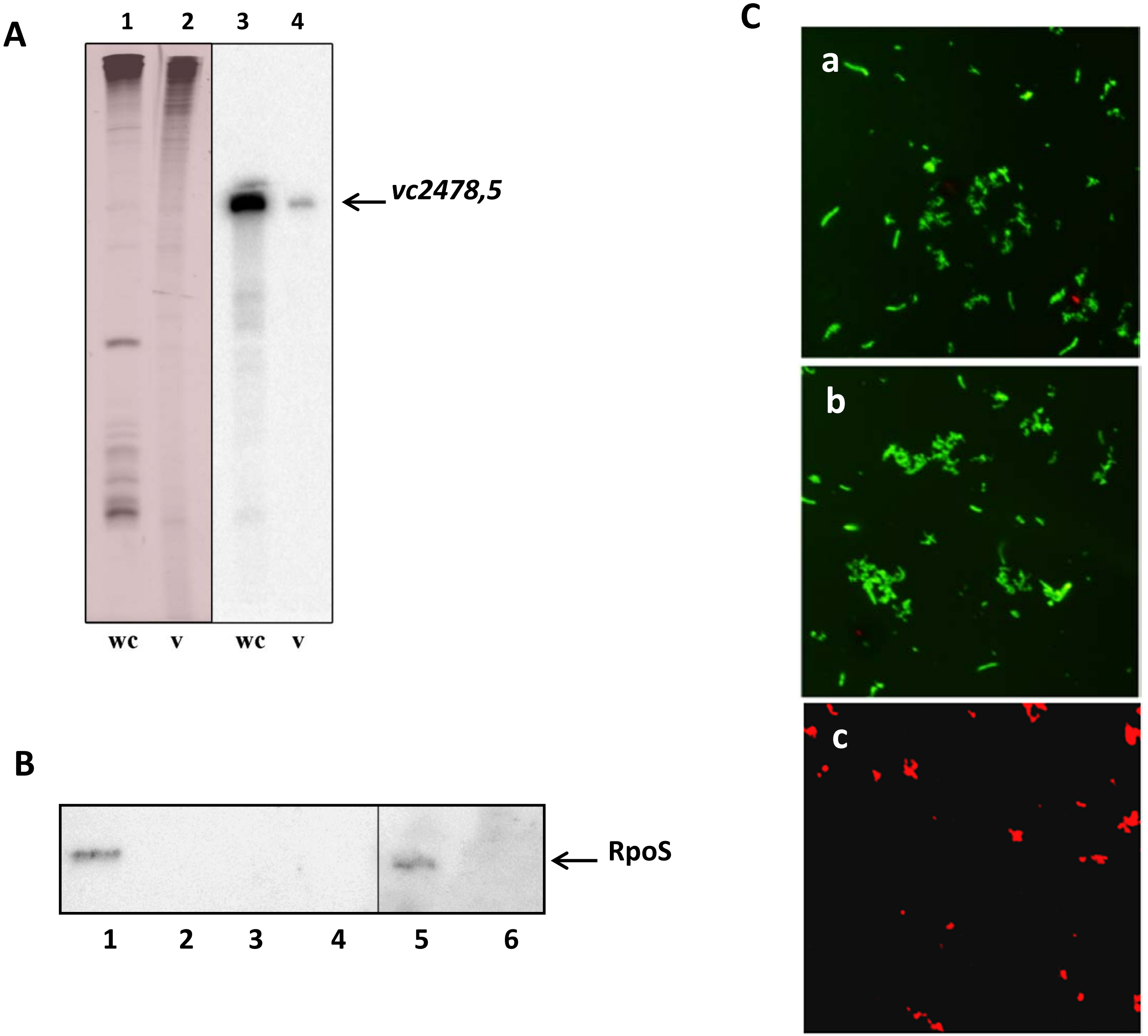

Supplementary Fig. S1

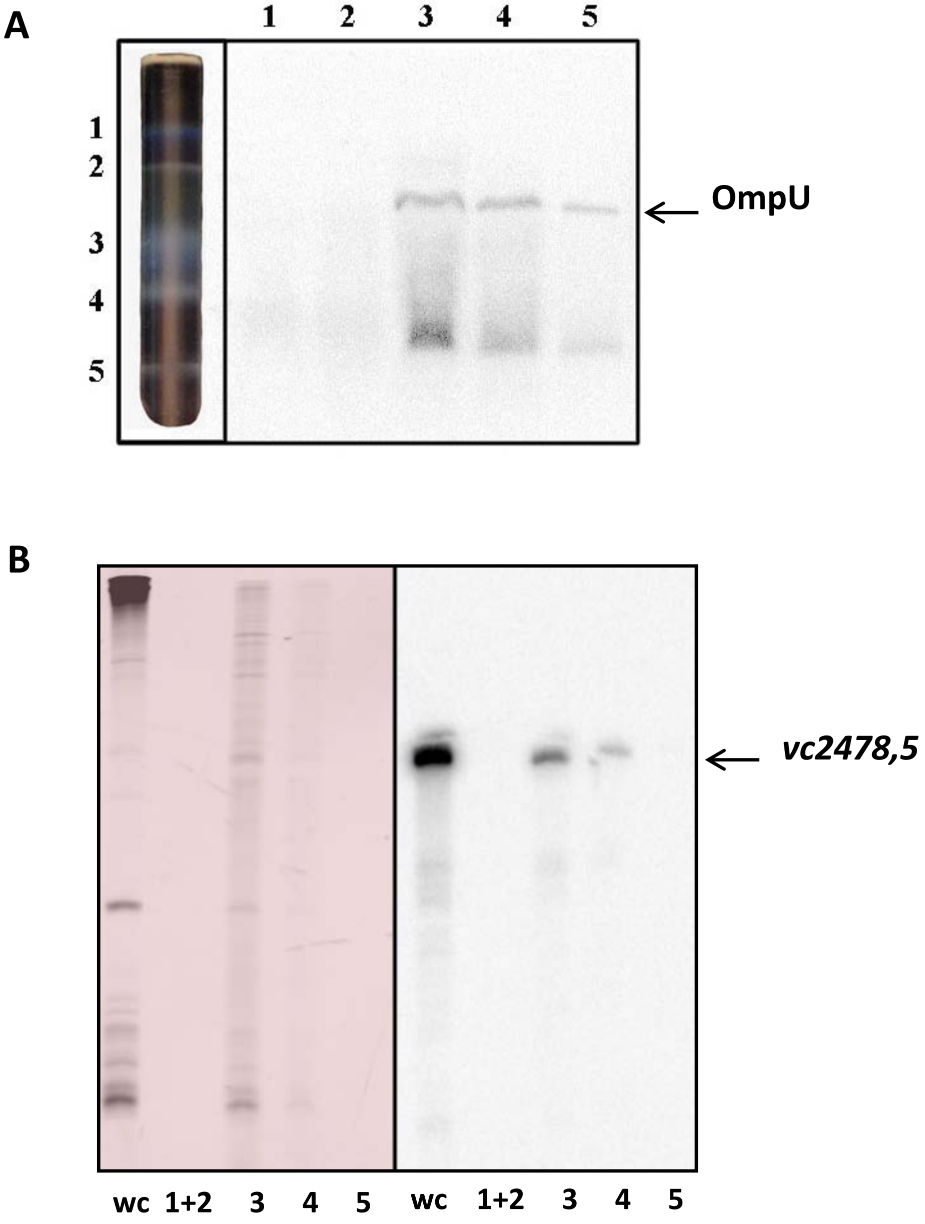

Supplementary Fig. S2

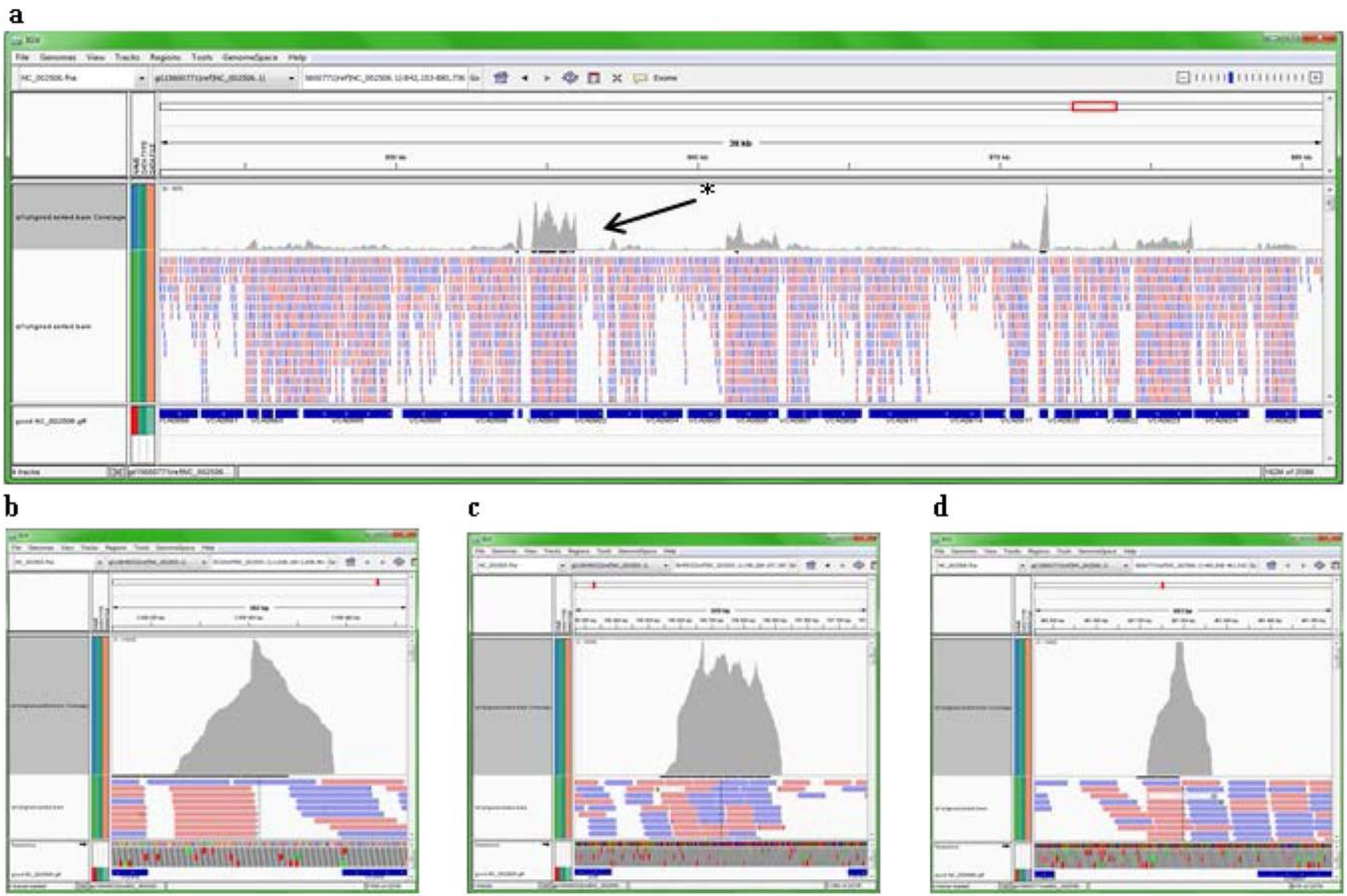

Supplementary Fig. S3

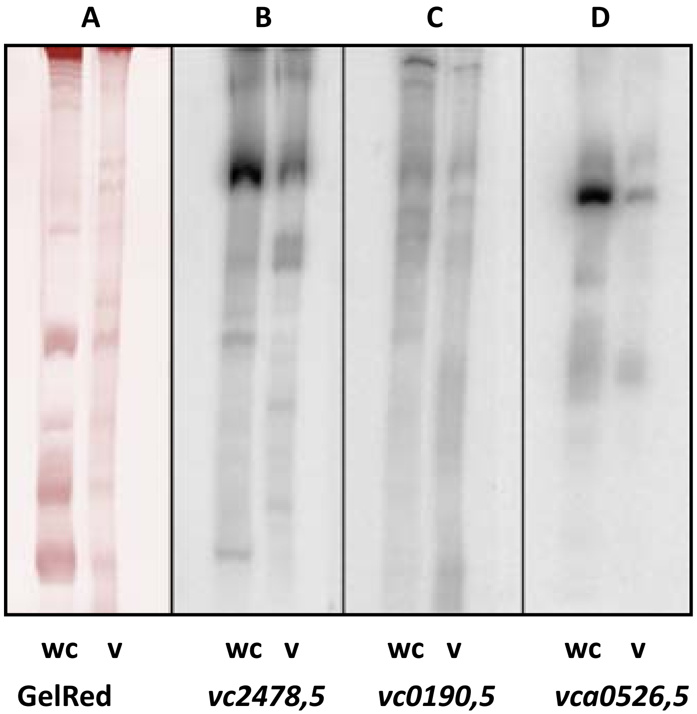

Supplementary Fig. S4

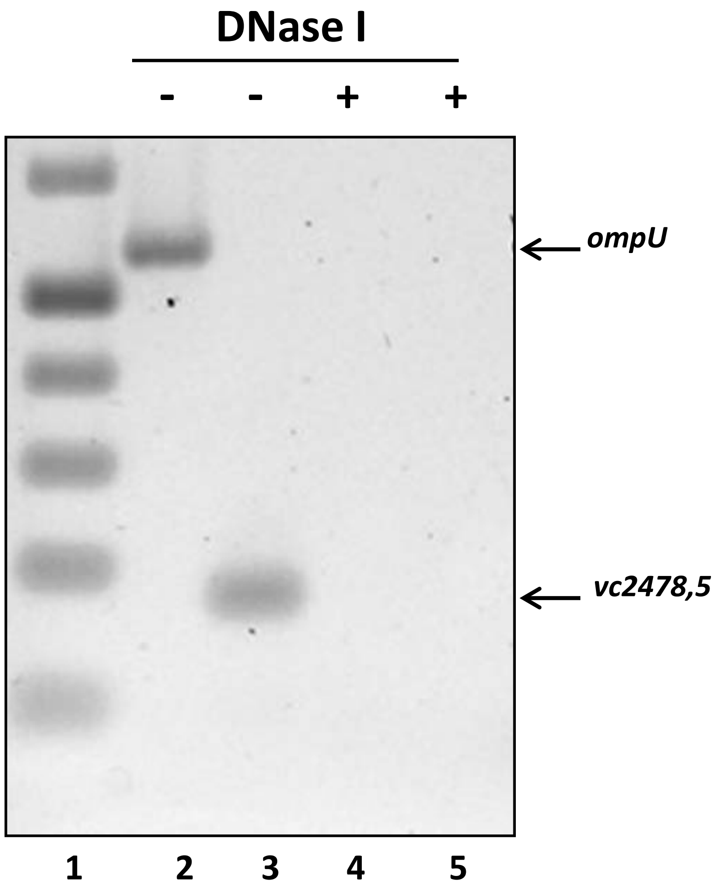

Supplementary Fig. S5

Supplementary Table 1a. Alignment of sequenced vesicle RNA to *V. cholerae* N16961 chromosome I.

**Peak**	**Start**	**End**	**Region in N16961 chromosome I**
**1090000**	2658225	2658386	upstream of uncharacterized protein **vc2479** **6S RNA**
**120000**	196545	196933	intergenic region between **vc0190** and **vc0191** **CsrB**
**111000**	914315	914654	upstream of phage integrase family **vc0847** **SrsA**
**41500**	943857	944200	uncharacterized protein **vc0883** **CsrB**
**41200**	2375908	2376115	uncharacterized protein **vc2221**
**37800**	2580714	2581110	upstream of uncharacterized protein **vc2410** svch2581.1
**23500**	1588979	1589272	ribosome modulating factor **vc1484**
**17800**	69300	69684	multidrug resistant protein **vc0069**
**13000**	2334461	2334692	downstream of flagellin A **vc2188**
**12100**	954751	954916	uncharacterized protein **vc0895** **GcvB**
**9400**	36506	36828	uncharacterized protein **vc0038**
**7300**	1578748	1578913	intergenic region between **vc1470** and **vc1471** svch1579.1 & 2
**7150**	1584873	1585042	intergenic region between **vc1478** and **vc1479**
**6700**	2619094	2619268	uncharacterized protein **vc2440**
**6200**	381650	382992	elongation factor Tu-B **vc0362**
**5600**	1719935	1720319	uncharacterized protein **vc1605a**
**5480**	1992699	1993856	putative porin **vc1854**
**5100**	1185676	1185924	putative glutaredoxin **vc1116**
**5000**	335290	336538	elongation factor Tu-A **vc0321**
**4300**	612554	613240	pseudo **vc0583** svch613.1 & 2
**4000**	1300189	1300577	integration host factor subunit alfa **vc1222**
**3700**	674802	675992	outer membrane protein **vc0633**
**3280**	1471635	1471788	uncharacterized protein **vc1378**
**3100**	2956240	2956682	ATP synthase F0F1 subunit I **vc2771**
**3010**	2174106	2174453	acyl carrier protein **vc2020**
**2930**	2332957	2334180	flagellin C **vc2187**
**2580**	1318751	1319201	uncharacterized protein **vc1247**
**2280**	2517106	2517539	autonomous glycyl radical cofactor **vc2361**
**2260**	2365289	2366647	outer membrane protein **vc2213**
**2190**	762615	762798	uncharacterized protein **vc0713**
**2000**	1538123	1539689	cbb-3 type Cytochrome c oxidase, subunit II **vc1442**
**1980**	1417217	1417374	upstream of uncharacterized protein **vc1333**
**1970**	222697	222833	coenzyme A biosynthesis bifunctional protein **vc0215**
**1940**	2063226	2063618	integration host factor subunit beta **vc1914**
**1910**	2295417	2295764	flagellar capping protein **vc2140**
**1880**	2754248	2755599	preprotein translocase subunit **vc2576**
**1800**	1408994	1410076	galactoside ABC transporter, periplasmic D-galactose/D-glucose-binding protein **vc1325**
**1780**	102446	102657	putative uncharacterized protein **vc0107** **RyhB & Spot_42**
**1750**	1198128	1198673	DNA binding protein **vc1130**
**1690**	1211912	1212296	cold shock-like protein **vc1142**
**1680**	2295764	2296334	flagellar protein **vc2141**
**1650**	2068862	2069279	DNA-binding protein HU-beta **vc1919**

**1650**	2909984	2911682	Phosphoenolpyruvate carboxykinase [ATP] **vc2738**
**1600**	1458356	1458653	uncharacterized protein **vc1368**
**1500**	86471	87681	cytochrome peroxidase c551 **vc0089**
**1490**	1670845	1673232	catalase/peroxidase **vc1560**
**1480**	1794805	1795288	heat shock protein **vc1663**
**1470**	2163539	2163748	uncharacterized protein **vc2009**
**1400**	786571	787319	antioxidant ahp CTSA family protein **vc0731**
**1390**	1536322	1537322	cbb-3 type Cytochrome c oxidase, subunit P **vc1439**
**1390**	2949663	2950113	ATP synthase F0F1 subunit epsilon **vc2763**
**1370**	2713024	2713375	RNA polymerase factor sigma-54 **vc2529**
**1330**	1537495	1538123	cbb-3 type Cytochrome c oxidase, subunit II **vc1441**
**1330**	2448193	2449428	Na(+)-translocating NADH-quinone reductase subunit **vc2294**
**1260**	1537322	1537495	cbb-3 type Cytochrome c oxidase, subunit Q **vc1440**
**1260**	2152257	2153283	glyceraldehyde-3-phosphate dehydrogenase **vc2000**
**1250**	2249553	2250272	succinate dehydrogenase iron-sulfur subunit **vc2088**
**1240**	580419	580787	upstream of and the whole carbon storage regulator **vc0548**
**1230**	1644504	1644628	intergenic region between **vc1531** and **vc1532**
**1200**	1097620	1099948	zink/cadmium/mercury/lead-transporting ATPase **vc1033**
**1190**	2449428	2450823	Na(+)-translocating NADH-quinone reductase subunit A **vc2295**
**1180**	2221154	2221925	flagellar biosynthesis sigma factor **vc2066**
**1140**	1031609	1031884	phosphocarrier protein HPr **vc0966**
**1140**	2394692	2394907	downstream of cytochrome c554 **vc2241**
**1140**	2669986	2670098	upstream of 2-isopropylmalate synthase **vc2490** svch2670.1
**1120**	1597539	1602466	uncharacterized protein **vc1492**
**1100**	1029112	1029661	PTS system glucos-specific IIA component **vc0964**
**1100**	2657848	2658199	uncharacterized protein **vc2478**
**1090**	1838582	1838921	uncharacterized protein **vc1707**
**1070**	564258	564667	lipoprotein **vc0533**
**1070**	2152042	2152257	putative uncharacterized protein **vc1999**
**1070**	2564959	2565969	cell division protein **vc2397**
**1060**	2564178	2564959	UDP-3-O-[3-hydroxymyristoyl] N-acetylglucosamine deacetylase **vc2396**
**1050**	2166629	2168114	PTS system glucose-specific transporter subunit IIBC **vc2013**
**1020**	2297656	2298854	flagellin D **vc2143**
**1000**	2296334	2297482	flagellin B **vc2142**
**1000**	462204	463183	malate dehydrogenase **vc0432**
**1000**	1520426	1520674	intergenic region between **vc1422** and **vc1423** svch1521.1
**1000**	1111516	1112882	long-chain fatty acid transport protein **vc1043**
**1000**	756233	756588	sigma-54 modulation protein **vc0706**
**980**	2253168	2254463	type II citrate synthase **vc2092**
**950**	2420154	2420290	upstream of uncharacterized protein **vc2264**
**950**	1310940	1311270	upstream of nicotinate-nucleotide--dimethylbenzimidazole phosphoribosyltransferase **vc1237**
**930**	2654106	2654548	uncharacterized protein **vc2473**
**920**	2019596	2019906	SpoVR family protein **vc1874**
**920**	1447024	1447739	uncharacterized membrane protein **vc1358**
**910**	2394907	2395221	cytochrome c554 **vc2241**
**910**	1650724	1651517	uncharacterized protein **vc1539** svch1651.1
**890**	2252361	2252772	succinate dehydrogenase cytochrome b556 subunit **vc2091**

**870**	1214761	1215323	ATP-dependent Clp protease ATP-binding subunit **vc1144**
**870**	1608509	1609145	solute/DNA competence effector, protein ProQ homolog **vc1497**
**870**	76886	77365	ferritin **vc0078**
**860**	305064	307130	acetyl-CoA synthetase **vc0298**
**850**	2071844	2073125	ATP-dependent protease ATP-binding subunit **vc1921**
**830**	2864880	2866417	aspartate ammonia-lyase **vc2698**
**830**	278425	278694	DNA binding HU-alfa **vc0273**
**820**	2444902	2446158	Na(+)-translocating NADH-quinone reductase subunit **vc2290**
**810**	1021356	1022017	uncharacterized protein **vc0957**
**810**	1814878	1815035	peptide ABC transporter substrate-binding protein **vc1680**
**810**	2764786	2765042	upstream of **vc2597** svch2765.1
**800**	1832388	1832689	uncharacterized protein **vc1699**
**800**	76031	76651	universal stress protein A **vc0076**
**780**	139954	140888	RNA polymerase sigma factor-32 **vc0150**
**770**	2713375	2713710	sigma-54 modulation protein **vc2530**
**760**	435431	435989	MSHA pilin protein **vc0409**
**740**	1451138	1452295	amino acid ABC transporter substrate binding protein **vc1362**
**740**	1158698	1160377	oligopeptide ABC transporter substrate binding protein **vc1091**
**730**	633774	636488	bifunctional aconitate hydratase 2/2 methylisocitrate **vc0604**
**730**	2081097	2082120	C4-dicarboxylate-binding periplasmic protein **vc1929**
**720**	2834286	2834637	co-chaperonin, 10 kDa chaperonin 1 **vc2665**
**720**	1135448	1135547	upstream of ArsR family transcriptional regulator **vc1068** svch1136.1 & 2
**720**	2178616	2179044	uncharacterized protein **vc2026**
**700**	2955395	2956240	ATP synthase F0F1 subunit A **vc2770**
**700**	2447420	2448193	Na(+)-translocating NADH-quinone reductase subunit **vc2293**
**700**	383504	384000	bactoferritin **vc0365**
**700**	132719	133283	DPS family protein **vc0139**
**700**	554239	554693	uncharacterized protein **vc0519**
**700**	2014929	2016993	uncharacterized protein **vc1872**
**680**	2954571	2955048	ATP synthase F0F1 subunit B **vc2768**
**680**	2446782	2447420	Na(+)-translocating NADH-quinone reductase subunit **vc2292**
**670**	692148	692445	polyribonucleotide nucleotidyltransferase **vc0647**
**670**	2877100	2878819	putative Sodium/solute symporter **vc2705**
**650**	2626588	2627926	enolase **vc2447**
**630**	2832601	2834286	molecular chaperone **vc2664**
**630**	1332776	1332950	DNA gyrase subunit A **vc1258**
**630**	2203125	2203796	superoxide dismutase **vc2045** svch2204.1
**620**	380939	381650	elongation factor G1 **vc0361**
**620**	500283	500485	S-adenosylmethionine synthetase **vc0472**
**610**	1319201	1320911	methyl-accepting chemotaxis protein **vc1248**
**600**	2950113	2951534	ATP synthase F0F1 subunit beta **vc2764**
**600**	2495392	2497494	elongation factor G 2 **vc2342**
**600**	2501622	2501760	intergenic region inbetween **vc2344** and **vc2345**
**590**	2250272	2252031	succinate dehydrogenase flavoprotein subunit **vc2089**
**590**	2450823	2450982	upstream of Na(+)-translocating NADH-quinone reductase subunit **vc2295**
**590**	2892438	2892948	uncharacterized protein **vc2717**
**580**	2347213	2348056	chemotaxis protein methyltransferase 1 **vc2201**

**560**	1374791	1375004	upsteam of phosphomethylpyrimidine kinase **vc1296** **TPP riboswitch**
**560**	1321079	1321598	uncharacterized protein **vc1249**
**550**	2344123	2345436	flagellar hook protein **vc2197**
**550**	2187578	2190491	bifunctional acetaldehyde-CoA/alcohol dehydrogenase **vc2033**
**550**	1283771	1284105	ds-DNA mimic protein **vc1208**
**540**	1477000	1477430	uncharacterized protein **vc1384**
**540**	2041347	2041496	intergenic region inbetween and parts of uncharacterized proteins **vc1893** and **vc1894**
**530**	1523028	1524280	spermidine/putrescine ABC transporter, periplasmic spermidine/putrescine-binding protein **vc1425**
**530**	1257042	1257620	uncharacterized protein **vc1183**
**520**	1651869	1652043	uncharacterized protein **vc1539a**
**520**	2952435	2954047	ATP synthase F0F1 subunit alfa **vc2766**
**520**	623301	623784	DnaK suppressor protein **vc0596**
**520**	2334684	2335901	flagellin A **vc2188**
**510**	133961	134317	uncharacterized protein **vc0142**
**510**	2083993	2084133	uncharacterized protein **vc1932**
**510**	952618	952804	chemotaxis protein **vc0893**
**500**	2955048	2955395	ATP synthase F0F1 subunit C **vc2769**
**500**	2334692	2336031	flagellin B **vc2142**
**500**	317733	318109	thioredoxin **vc0306**
**490**	1300020	1300189	upstream of **vc1222**
**480**	2327636	2327793	4-diphosphocytidyl-2-C-methyl-D-erythritol kinase **vc2182**
**470**	2582987	2584882	pyruvate dehydrogenase **vc2413**
**470**	2951534	2952435	ATP synthase F0F1 subunit gamma **vc2765**
**470**	2414371	2415329	elongation factor Ts **vc2259**
**470**	2006853	2009215	formate acetyltrancferase **vc1866**
**470**	935777	935909	upstream of IS1004 transposase **vc0870**
**460**	2782151	2782911	cAMP-regulatory protein **vc2614**
**460**	107395	108097	cytochrome c4 **vc0112**
**450**	2783856	2784067	N-succinylglutamate 5-semialdehyde dehydrogenase **vc2616**
**450**	2954047	2954571	ATP synthase F0F1 subunit delta **vc2767**
**450**	2856763	2857292	6-phosphofructokinase **vc2689**
**450**	1976501	1977100	peptidoglycan-associated lipoprotein **vc1835**
**440**	1385378	1386948	putative fumarate hydratase, class I **vc1304**
**440**	2393494	2393849	nitrogen regulatory protein P-II **vc2239**
**430**	1530646	1531594	putative stress protein **vc1433**
**415**	2055506	2056621	alanine dehydrogenase **vc1905**
**410**	1050496	1051173	Adenylate kinase **vc0986**
**410**	77365	77729	universal stress protein B, ppGpp-dependent, membrane associated **vc0079**
**400**	2348056	2349080	chemotaxis protein **vc2202**
**400**	803130	803283	HesB family protein **vc0750**
**400**	1100574	1101161	uridine phosphorylase **vc1034**
**390**	354674	355141	2,3-bisphosphoglycerate-independent phosphoglycerate mutase **vc0336**
**390**	2130297	2130520	putative aspartate amino transferase **vc1977**
**390**	302338	302528	Acetyl-CoA carboxylase, biotin carboxylase **vc0295**
**390**	2513905	2514095	uncharacterized protein **vc2357**
**380**	2218550	2219998	chemotaxis protein **vc2063**
**380**	2610643	2611874	outer membrane channel protein **vc2436**

**370**	2944713	2945721	3-ketoacyl-CoA thiolase **vc2759**
**370**	2343586	2343966	flagellar basal-body rod protein **vc2196**
**370**	1801911	1802093	intergenic region between **vc1669** and **vc1670**
**350**	564667	565745	RNA polymerase sigma factor-38 **vc0534**
**350**	348760	349396	anti-RNA polymerase sigma 70 factor **vc0330**
**350**	96097	97811	methyl accepting chemotaxis protein **vc0098**
**340**	2581316	2582415	dihydrolipoyl dehydrogenase **vc2412**
**340**	2446158	2446782	Na(+)-translocating NADH-quinone reductase subunit **vc2291**
**330**	2524608	2525234	aerobic respiration control protein **vc2368**
**330**	2345436	2346180	basal-body rod modification protein **vc2198**
**330**	1407889	1408268	uncharacterized proteins **vc1322** and **vc1323**
**330**	2143461	2143732	uncharacterized protein **vc1991**
**320**	2584882	2587569	pyruvate dehydrogenase E1 component **vc2414**
**320**	792153	792676	putative Acetoin utilization protein **vc0737**
**320**	1381458	1382755	serine transporter **vc1301**
**320**	1396268	1396793	methyl-accepting chemotaxis protein **vc1313**
**320**	2799112	2799302	DamX-related protein **vc2627**
**310**	2214140	2214592	purine-binding chemotaxis protein **vc2059**
**310**	1336667	1336810	uncharacterized protein **vc1262**
**300**	2220725	2221154	chemotaxis protein **vc2065**
**300**	1021344	1021482	intergenic region between **vc0956** and **vc0957**
**290**	2263005	2263276	intergenic region between **vc2105** and **vc2106**
**280**	1114605	1114725	intergenic region between **vc1045** and **vc1046**
**280**	1061324	1062905	PTS systemN-acetylglucosamine-specific transporter subunit **vc0995**
**280**	2801124	2801281	fimbrial assembly protein **vc2630**
**280**	132624	132718	uncharacterized protein **vc0138**
**270**	665838	666197	essential respiratory protein A, iron-sulfur insertion prot **vc0627**
**270**	2346618	2347031	flagellar basal body rod protein **vc2200**
**270**	2221925	2222617	site-determining protein **vc2067**
**260**	2587569	2588098	pyruvate dehydrogenase complex repressor v**c2415**
**260**	53506	53590	uncharacterized protein **vc0059**
**250**	2349799	2350245	negative regulator of flagellin synthesis **vc2204**
**250**	2288320	2288626	flagellar hook-basal body complex protein **vc2134**
**250**	2627926	2628183	CTP synthase **vc2448**
**250**	422634	423163	single-stranded DNA-binding protein **vc0397**
**250**	2420288	2420723	UPF0325 protein **vc2264**
**250**	2252772	2252962	upstream of **vc2091**
**250**	937693	937804	uncharacterized protein **vc0874**
**240**	2549464	2549770	upstream of RNA-directed DNA polymerase **vc2385** **IGR3**
**230**	2839602	2840135	regulator of ribonuclease activity A **vc2672**
**220**	1401314	1402103	outer membrane protein **vc1318**

Peak: the highest number of RNA sequences aligned to a specific nucleotide the region. Grey box: region contains a sRNA. **Start** and **end**: positions in the chromosome. Orange box: cytoplasmic location, Blue box:

inner membrane location, Red box: outer membrane location, Purple box: membrane, Light yellow box: pilus association, Yellow box: flagellar association, and White box: unknown location.

Supplementary Table 1b. Alignment of sequenced vesicle RNA to *V. cholerae* N16961 chromosome II.

**Peak**	**Start**	**End**	**Region in N16961 chromosome II**	
**115300**	461118	461267	intergenic region between **vca0526** and **vca0527**	svch462.1
**92300**	783813	784124	uncharacterized protein **vca0839**	**CsrB**
**24200**	886914	887278	uncharacterized protein **vca0935**	
**19400**	66495	66837	major outer membrane lipoprotein **vca0059**	
**8400**	61091	61300	uncharacterized protein **vca0052**	
**7590**	1031448	1031574	upstream of LuxR family transcriptional regulator **vca1078**	
**7500**	308592	309077	translation initiation factor IF-3 **vca0288**	**LR-PK1**
**7000**	485524	485763	uncharacterized protein **vca0547**	svch486.1
**5800**	213120	213285	upstream of GMP reductase **vca0197**	svch214.1
**4650**	893294	893459	downstream of maltose transport system permease protein **vca0943**	**MicX**
**4220**	817334	819261	hemagglutinin/protease **vca0865**	
**3090**	819602	820290	outer membrane protein **vca0867**	
**2700**	284969	285940	methyl-accepting chemotaxis protein **vca0268**	
**2180**	627734	628099	uncharacterized protein **vca0689**	
**2180**	893459	893603	maltose transport system permease protein **vca0943**	svch894.2
**1670**	834562	835685	uncharacterized protein **vca0883**	
**1600**	4061	4387	uncharacterized protein **vca0004**	
**1430**	308245	308592	upstream of translation initiation factor IF-3 **vca0288**	
**1370**	487754	488020	uncharacterized protein **vca0551**	
**1140**	203421	203797	cold shock DNA-binding domain-containing protein **vca0184**	
**1100**	39233	40071	upstream of and uncharacterized protein **vca0032**	
**1020**	232124	232329	upstream of uncharacterized protein **vca0213**	svch233.1
**1010**	994761	994875	upstream of PTS system mannitol-specific EIICBA component **vca1045**	**MtlS**
**950**	88629	89022	uncharacterized protein **vca0078**	
**830**	776851	776956	uncharacterized protein **vca0831**	
**660**	629320	630236	acetyl-CoA reductase **vca0691**	
**630**	833061	833490	uncharacterized protein **vca0881**	
**610**	628099	629320	acetyl-CoA acetyltransferase **vca0690**	
**580**	971762	972073	uncharacterized protein **vca1024**	
**560**	532373	532660	hemolysin **vca0594**	
**550**	237171	237395	upstream of hemolysin **vca0219**	
**530**	220268	220397	pseudo **vca0203**	
**510**	684347	685544	uncharacterized protein **vca0738**	
**500**	508610	509011	uncharacterized protein **vca0571**	
**500**	871320	871655	uncharacterized protein **vca0919**	
**500**	929290	929858	uncharacterized protein **vca0981**	
**490**	1003717	1004369	uncharacterized protein **vca1054**	
**480**	546334	546515	uncharacterized protein **vca0609**	
**450**	556852	558082	upstream of and transaldolase **vca0623**	svch557.1
**430**	702687	702937	arginine ABC transporter, permease protein **vca0758**	
**420**	833490	834562	uncharacterized protein **vca0882**	
**410**	678373	678819	uncharacterized protein **vca0732**	
**400**	89052	90407	uncharacterized protein **vca0079**	
**400**	204597	205022	uncharacterized protein **vca0186**	
**400**	760422	760987	agmatinase **vca0814**	

**390**	292709	292829	upstream of IS1004 transposase **vca0275**
**390**	854501	855376	uncharacterized protein **vca0900**
**370**	38714	39233	methyl-accepting chemotaxis protein **vca0031**
**370**	508251	508610	translation initiation factor Sui1 **vca0570**
**360**	1050275	1050461	chemotaxis protein **vca1095**
**360**	1052862	1053168	uncharacterized protein **vca1097**
**350**	1068928	1069971	spermidine/putrescine ABC transporter, periplasmic spermidine/putrescine-binding protein **vca1113**
**340**	1029148	1029441	uncharacterized protein **vca1075**
**330**	713202	713355	uncharacterized protein **vca0769**
**320**	42217	43058	uncharacterized protein **vca0034**
**320**	760210	760373	intergenic region between **vca0813** and **vca0814**
**320**	601944	602091	probable anaerobic C4-dicarboxylate transporter **vca0665**
**310**	787274	787400	downstream of glyceraldehyde 3-phosphate dehydrogenase **vca0844**
**310**	86599	86766	sulfate permease family protein **vca0077**
**300**	471351	471626	Sec-independent protein translocase protein **vca0533**
**300**	1068580	1068928	tRNA(Met) cytidine acetyltransferase **vca1112**
**290**	295897	296280	glycine cleavage system H protein **vca0277**
**290**	855376	856001	uncharacterized protein **vca0901**
**280**	184104	184447	cold shock transcriptional regulator **vca0166**
**280**	483866	484022	upstream of uncharacterized protein **vca0546** svch484.1
**280**	750895	751260	uncharacterized protein **vca0806**
**280**	1049376	1049696	purine-binding chemotaxis protein **vca1093**
**270**	1026467	1026730	bifunctional proline dehydrogenase/pyrroline-5-carboxylate dehydrogenase **vca1073**
**270**	637183	637351	upstream of Glucose-1-phosphate adenylyltransferase 2 **vca0699**
**260**	993053	993422	Ccm2-related protein **vca1042**
**260**	897542	897683	intergenic region between **vca0945** and **vca0946**
**260**	969225	969635	uncharacterized protein **vca1021**
**250**	854000	854194	uncharacterized protein **vca0899**
**240**	194828	195055	uncharacterized protein **vca0177**
**240**	531212	532265	uncharacterized protein **vca0593**
**240**	1021264	1021692	sodium/proline symporter **vca1071**
**240**	1022765	1023456	uncharacterized protein **vca1072**
**230**	333994	334479	uncharacterized protein **vca0332**
**230**	580379	580807	uncharacterized protein **vca0645**
**230**	876133	876422	methyl-accepting chemotaxis protein **vca0923**
**220**	860933	861670	methyl-accepting chemotaxis protein **vca0906**
**220**	832592	833042	uncharacterized protein **vca0880**
**210**	180156	180453	upstream of tryptophanase **vca0161**

Peak: the highest number of RNA sequences aligned to a specific nucleotide the region. Grey box: region contains a sRNA. **Start** and **end:** positions in the chromosome. Orange box: cytoplasmic location, Red box: outer membrane location, Blue box: inner membrane location, Light blue box: periplasmic location, Purple box: membrane, Green box: secreted protein, and White box: unknown location.

Supplemental References

Amako, K., Meno, Y., and Takade, A. (1988). Fine structures of the capsules of *Klebsiella pneumoniae* and *Escherichia coli* K1. J. Bacteriol. *170*, 4960–4962.

Benson, S.A., and Decloux, A. (1985). Isolation and characterization of outer membrane permeability mutants in *Escherichia coli* K-12. J. Bacteriol. *161*, 361–367.

Bolivar, F., Rodriguez, R.L., Greene, P.J., Betlach, M.C., Heyneker. H.L., Boyer, H.W., Crosa, J.H., and Falkow, S. (1977). Construction and characterization of new cloning vehicles. II. A multipurpose cloning system. Gene *2*: 95–113.

Casadaban, M.J. (1976). Transposition and fusion of the *lac* genes to selected promoters in *Escherichia coli* using bacteriophage lambda and Mu. J. Mol. Biol. *104*, 541–555.

Chatterjee, S., Mondal, A.K., Begum, N.A, Roychoudhury, S., and Das, J. (1998). Ordered cloned DNA map of the genome of *Vibrio cholerae* 569B and localization of genetic markers. J. Bacteriol. *180*, 901–908.

Furste, J.P., Pansegrau, W., Frank, R., Blocker, H., Scholz, P., Bagdasarian, M., and Lanka, E. (1986). Molecular cloning of the plasmid RP4 primase region in a multi-host-range *tacP* expression vector. Gene. 48, 119–31.

Guzman, C.A., Piatti, G., Staendner, L.H., Biavasco, F., and Pruzzo, C. (1995) Export of *Bordetella pertussis* serotype 2 and 3 fimbrial subunits by *Escherichia coli*. FEMS Microbiol. Lett. *128*, 189–194.

Miller, V.L., Taylor, R.K., and Mekalanos, J.J. (1987). Cholera toxin transcriptional activator *toxR* is a transmembrane DNA binding protein. Cell *48*, 271–279.

Taylor, R.K., Miller, V.L., Furlong, D.B., and Mekalanos, J.J. (1987). Use of *phoA* gene fusions to identify a pilus colonization factor co-coordinately regulated with cholera toxin. Proc. Natl. Acad. Sci. USA *84*, 2833–2837.

Thelin, K.H., and Taylor, R.K. (1996). Toxin-coregulated pilus, but not mannose-sensitive hemagglutinin, is required for colonization by *Vibrio cholerae* O1 El Tor biotype and O139 strains. Infect. Immun. *64*, 2853–2856.

Yildiz, F.H., and Schoolnik, G.K. (1998). Role of rpoS in stress survival and virulence of *Vibrio cholerae*. J. Bacteriol. *180*, 773–784.
